# KIAA1429 increases FOXM1 expression through YTHDF1–mediated m6A modification to promote aerobic glycolysis and tumorigenesis in multiple myeloma

**DOI:** 10.1007/s10565-024-09904-2

**Published:** 2024-07-26

**Authors:** Yue Wu, Yi Luo, Xingchen Yao, Xiangjun Shi, Ziyu Xu, Jie Re, Ming Shi, Meng Li, Junpeng Liu, Youzhi He, Xinru Du

**Affiliations:** 1 Department of Orthopedics, Beijing Chao-Yang Hospital, No.8 Gongti South Rd, Chaoyang District, Beijing, 100020 China; 2 Department of Spine Surgery, Hengyang Medical School, The Affiliated Changsha Central Hospital, University of South China, Changsha, 410007 Hunan China

**Keywords:** Multiple myeloma, KIAA1429, YTHDF1, FOXM1, m6A, Aerobic glycolysis

## Abstract

**Objective:**

Multiple myeloma (MM) is a deadly plasma cell malignancy with elusive pathogenesis. N6-methyladenosine (m6A) is critically engaged in hematological malignancies. The function of KIAA1429, the largest component of methyltransferases, is unknown. This study delved into the mechanism of KIAA1429 in MM, hoping to offer novel targets for MM therapy.

**Methods:**

Bone marrow samples were attained from 55 MM patients and 15 controls. KIAA1429, YTHDF1, and FOXM1 mRNA levels were detected and their correlation was analyzed. Cell viability, proliferation, cell cycle, and apoptosis were testified. Glycolysis-enhancing genes (HK2, ENO1, and LDHA), lactate production, and glucose uptake were evaluated. The interaction between FOXM1 mRNA and YTHDF1, m6A-modified FOXM1 level, and FOXM1 stability were assayed. A transplantation tumor model was built to confirm the mechanism of KIAA1429.

**Results:**

KIAA1429 was at high levels in MM patients and MM cells and linked to poor prognoses. KIAA1429 knockdown restrained MM cell viability, and proliferation, arrested G0/G1 phase, and increased apoptosis. KIAA1429 mRNA in plasma cells from MM patients was positively linked with to glycolysis-enhancing genes. The levels of glycolysis-enhancing genes, glucose uptake, and lactate production were repressed after KIAA1429 knockdown, along with reduced FOXM1 levels and stability. YTHDF1 recognized KIAA1429-methylated FOXM1 mRNA and raised FOXM1 stability. Knockdown of YTHDF1 curbed aerobic glycolysis and malignant behaviors in MM cells, which was nullified by FOXM1 overexpression. KIAA1429 knockdown also inhibited tumor growth in animal experiments.

**Conclusion:**

KIAA1429 knockdown reduces FOXM1 expression through YTHDF1-mediated m6A modification, thus inhibiting MM aerobic glycolysis and tumorigenesis.

**Graphical Abstract:**

KIAA1429 knockdown reduces FOXM1 expression through YTHDF1-mediated m6A modification, thus inhibiting aerobic glycolysis and tumorigenesis in MM

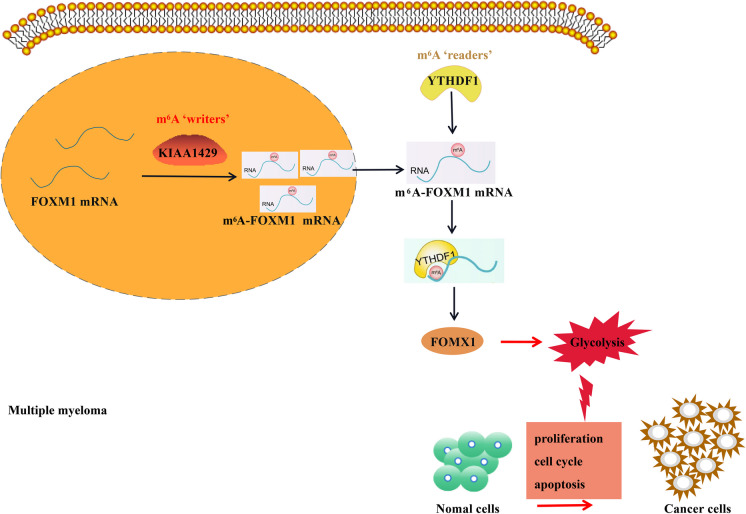

**Supplementary Information:**

The online version contains supplementary material available at 10.1007/s10565-024-09904-2.

## Introduction

Multiple myeloma (MM) is a fatal plasma cell (PC) malignancy that takes up nearly 10% of hematological malignancies, with the incidence increased every year (Medical Masterclass & Firth 2019; Yang et al. 2020). In spite of advancements in MM diagnosis and treatment, the clinical outcome has not yet been satisfactory (McCullough et al. 2018; Naymagon & Abdul-Hay 2016). Owing to the complex pathogenesis, the detailed underlying mechanisms of MM development remain largely unknown, making it an incurable to some extent. Thereby, it is pressingly critical to identify its pathogenic mechanisms.

N6-methyladenosine (m6A) is the most plentiful RNA modification (Zhao et al. 2020). In eukaryotes, m6A modifications has multiple functions such as mRNA stabilization, export, splicing, and translation (Shi et al. 2017; Wang et al. 2015; Xiao et al. 2016). Methyltransferases encompass METTL3, WTAP, KIAA1429, and METTL14 (Roundtree et al. 2017). Increasing evidence suggests important roles of m6A machinery in hematological malignancies, including MM (Zhao & Peng 2022). Multiple studies have elucidated the regulatory roles of m6A writer METTL3 (Che et al. 2023), m6A eraser FTO (Xu et al. 2022), and ALKBH5 (Qu et al. 2022; Yu et al. 2022), and m6A readers HNRNPA2B1 (Jiang et al. 2021; Liu et al. 2022), and YTHDF2 (Hua et al. 2022; Liu et al. 2023) in MM progression. Biochemical studies have shown that KIAA1429 recruits METTL14/WTAP/METTL3 to RNA substrates for m6A modification (Yue et al. 2018). Nonetheless, the role of KIAA1429 in MM is unclarified.

Cancer cells take up more glucose than normal tissues and metabolize glucose through glycolysis (an inefficient pathway for ATP production), a process now recognized as aerobic glycolysis (Warburg effect), which is a typical quality of cancer cells and is critical for their growth (Liberti & Locasale 2016). Aerobic glycolysis influences tumor progression by participating in cancer cell biosynthesis, influencing the tumor microenvironment and tumor cell signaling (Liberti & Locasale 2016). Aerobic glycolysis has gained increasing attention as a new therapeutic target in various cancers such as MM (Ganapathy-Kanniappan & Geschwind 2013; Gu et al. 2017; Sanchez et al. 2013; Stine et al. 2022). Studies have denoted the involvement of KIAA1429 in aerobic glycolysis regulation in colorectal cancer (Li et al. 2022) and gastric cancer (Yang et al. 2021). Whether it may be involved in MM by promoting the aerobic glycolysis has not yet been reported.

m6A methyltransferases can stabilize mRNAs of members of the FOXO family, including FOXM1 (Jian et al. 2020; Tang et al. 2023) in a wide range of diseases. FOXM1 can regulate glycolysis in MM (Cheng et al. 2022). YTH m6A RNA-binding protein (YTHDF1) is a powerful "reader" protein for m6A methyltransferases and is involved in disease processes by enhancing the stability of target RNAs (Chen et al. 2021). For example, YTHDF1 accelerates FOXM1 translation and drives breast cancer (BC) metastasis through m6a modification (Chen et al. 2022). YTHDF1 is recruited by KIAA1429 to the transcription factor FOXM1 to stabilize FOXM1 mRNA and thus regulate cisplatin sensitivity in gastric cancer cells (Zhu et al. 2022). Importantly, evidence also notes the involvement of YTHDF1 in regulating glycolysis in BC (Yao et al. 2022). It remains uncertain whether KIAA1429 can stabilize FOXM1 expression via YTHDF1-mediated m6A, and thus promoting MM aerobic glycolysis and tumorigenesis, has not yet been reported. Here, we delved into the mechanism of KIAA1429 in MM, with the aim of providing data for the search of possible new targets for MM therapy.

## Methods

### Sample collection

Bone marrow samples were acquired from 55 patients first diagnosed with MM in Beijing Chao-Yang Hospital from 2018.1–2020.12. Bone marrow samples were harvested from 15 healthy subjects matched to the gender and age of MM patients who underwent orthopaedic surgery in Beijing Chao-Yang Hospital during the same period as controls. Referring to previous research (Lin et al. 2004; Rawstron et al. 2008), flow cytometry was used to identify isolated PCs (MM–PCs) derived from MM patients’ bone marrow and normal PCs (N-PCs) derived from healthy subjects' bone marrow using specific light scattering distribution and reaction patterns of CD138, CD38, and CD45. The purity of the obtained MM-PCs and N-PCs was more than 95%. In addition, all MM patients were followed up until December 2022, and 17 patients were lost. The lost cases were recorded with the last clear survival time, while those who survived the last follow-up were recorded with truncated data to analyze overall survival (OS), from diagnosis to death or last follow-up.

All participants offered written informed consent and experiments followed the Helsinki Declaration. This paper was ratified by our Ethics Committee.

### Cell culture

Human MM cell lines NCI-H929, MM.1S, U266, RPMI8226, and CAG (procured from ATCC, USA) were grown in RPMI-1640 plus 10% FBS, penicillin (10,000 U/L), and streptomycin (100 mg/L) at 37 °C/5% CO_2_. N-PCs derived from bone marrow of healthy subjects were used as controls.

### Cell transfection

The control plasma pLK0.1-puro and shRNAs targeting KIAA1429 and YTHDF1 were procured from Sigma (MO, USA). ShRNA plasmid was cotransfected with packaging structure lentivirus for 48 hs. NCI-H929/MM.1S was cotransfected with YTHDF1 shRNA (50 nM) and lentivirus vector carrying pcDNA3.1-FOXM1 (oe–FOXM1, 10 nM) or pcDNA3.1-NC (oe NC) using Lipofectamine 2000 (Invitrogen, USA).

### RT-qPCR

RNA was extracted from N-PCs or human MM cells with TRIzol, followed by measurement of purity and concentration of extracted RNAs on NanoDrop 2000 and synthesis of cDNA using the FastQuant RT Kit. Afterward, SYBR Premix Ex TaqII (Takara) was applied for RT-qPCR using the ABI7500 system. Using GAPDH as a parameter, the 2-^ΔΔCt^ method was applied for data calculation (Schmittgen & Livak 2008). These primers (displayed in Table 1) were synthesized by Sangon Biotech.

**Table 1:** Primer sequences for RT–qPCR

Gene	Forward 5’-3’	Reverse 5’-3’
KIAA1429	AAGTGCCCCTGTTTTCGATAG	ACCAGACCATCAGTATTCACCT
HK2	CAAAGTGACAGTGGGTGTGG	GCCAGGTCCTTCACTGTCTC
ENO1	TGCGTCCACTGGCATCTAC	CAGAGCAGGCGCAATAGTTTTA
LDHA	ATCTTGACCTACGTGGCTTGGA	CCATACAGGCACACTGGAATCTC
FOXM1	TCTGCCAATGGCAAGGTCTCCT	CTGGATTCGGTCGTTTCTGCTG
YTHDF1	CAGCACCGATCCCGACATAG	CTGGCTTCCTGAAGACGATGA
GAPDH	AATCCCATCACCATCTTCCAG	AAATGAGCCCCAGCCTTC

### Western blot

Total proteins were extracted from human MM cells with RIPA lysis buffer and quantified by the BCA method. Briefly, proteins were subjected to electrophoretic separation in SDS-PAGE and moved onto PVDF membranes, followed by blockade for 1 h with 5% non-fat milk in TBST and overnight incubation with diluted primary antibodies (1:1000) [KIAA1429 (ab271136, Abcam), FOXM1 (ab207298, Abcam), YTHDF1 (ab220162, Abcam), and β-actin (the control, 1:5000, ab179467, Abcam)] at 4 °C. Subsequently, membranes were probed for 1 h with a secondary antibody IgG H&L (HRP) at ambient temperature, and protein bands were visualized.

### CCK–8 assay

Cell viability was testified by CCK–8 kits. Subsequent to incubation in 96-well plates (2 × 10^4^/well) at 37℃ for 24, 48, and 72 hs, cells were further incubated for 2 h with 10 μL CCK-8. Absorbance was determined at 450 nm by a spectrophotometer. Cell proliferation was examined using EdU kits (C103103, RiboBio) and cells were observed by a fluorescence microscope.

### Cell cycle measurement

After 24–hs incubation at 1 × 10^6^ cells/well in 6-well plates, cells were permeabilized at 4 °C with pre-cooled 75% ethanol overnight, cultured for 30 min with 1 mg/mL RNase A, and dyed for 15 min with 50 μg/mL PI in dark, followed by analysis on a FACSCalibur flow cytometer.

### Cell apoptosis measurement

Based on Annexin V-FITC/PE Apoptosis kit instructions, cells were rinsed in PBS, resuspended in a binding buffer (100 μL), and incubated for 5 min with Annexin V-FITC (5 μL) before 15-min incubation in dark with 5 μL PI. Fluorescence intensity was tested by flow cytometry.

### Glucose uptake detection

According to glucose uptake assay kit instructions, MM cells were rinsed thrice with PBS, starved by 40-min pre–incubation with 100 μL HEPES buffer, stimulated for 20 min with (or without) 1 μM insulin to activate glucose transporter, and cultured for 20 min with 10 μL 2-deoxyglucose (10 mM). After being lysed with an extraction buffer (90 μL), cells were frozen/thawed once and heated for 40 min at 85 °C. Next, cell lysate was neutralized by adding neutralization buffer (10 μL). Finally, glucose uptake was assessed with cellular fluorescence (Ex/Em = 535/587 nm).

### Lactate production assay

According to lactate assay kit (Biovision, USA) instructions, MM cells were grown in fresh phenol-free RPMI-1640 medium, which was obtained at indicated time and then mixed with lactate assay buffer in 96-well plates (50 μL/well). Next, reaction buffer was supplemented (50 μL/well) for 30-min incubation. Lactate production was assessed by the absorbance at 570 nm.

### RIP and MeRIP

RIP was performed to determine the YTHDF1-FOXM1 mRNA interaction. MeRIP was used to examine m6A on FOXM1 mRNA by Magna RIP Kits. MM cells were lysed in RIP lysis buffer. Before the addition of protein G dynabeads, cells were supplemented with 5 µg of m6A or YTHDF1 antibody at 4 °C overnight while rotation. Subsequent to elution with Proteinase K, RNA was purified with the RNeasy Mini Kit, and pulled down RNA was analyzed by RT-qPCR: FOXM1 motif, forward: 5’-CCTCTGAGTGAGGACAGCAG-3’, reverse: 5’-AACACAAGGTCCCAGCAGTG-3’.

### mRNA stability assay

NCI-H929 and MM.1S cells were grown at 3 × 10^5^/well in 6-well plates before infection with KIAA1429 or YTHDF1 shRNAs. Following 5 µg/mL Actinomycin D treatment, cells were gathered for total RNA extraction, and 1 µg total RNA was synthetized to cDNA, followed by detection of FOXM1 mRNA level by RT-qPCR.

### Animal experiments

Female BALB/c nude mice (4–6 weeks old, 16–20 g) from Vital River (Beijing, China) were kept in facilities without specific pathogens. In this study, we used a total of 12 mice for in vivo animal experiments. The 12 mice were randomized into 2 groups of 6 mice each. NCI-H929 cells delivered with sh-NC or sh-KIAA1429 lentivirus vector were injected into mice. Tumor volume was inspected every 3 days and computed: tumor volume = 0.5 × Long × Width^2^. Four weeks following cell injection or when the tumor diameter exceeded 15 mm, mice were killed and the tumor was resected for immunohistochemistry (IHC) (Li et al. 2021). Specifically, xenograft tumors were soaked in 4% formalin, embedded in paraffin, and sliced at 5 μm. After routine dewaxing, rehydration, antigen retrieval, and blockade, sections were probed overnight with specific antibodies at 4 °C and with a secondary antibody. Finally, the results were observed by an Olympus microscope.

### Statistical analysis

GraphPadPrism8.01 software was adopted for data processing. Measurement data in normal distribution confirmed by Shapiro Wilk test were depicted as mean ± SD. Pairwise comparisons were done by t test, and multi-groups comparisons were done by one-way ANOVA, with Tukey’s test for post-test. The Kaplan–Meier method was carried out for analyzing the relationship between KIAA1429 and the prognosis of MM patients. *P* < 0.05 implied statistical significance.

## Results

KIAA1429 is raised in MM and correlated with miserable prognosis.

**Fig. 1:**
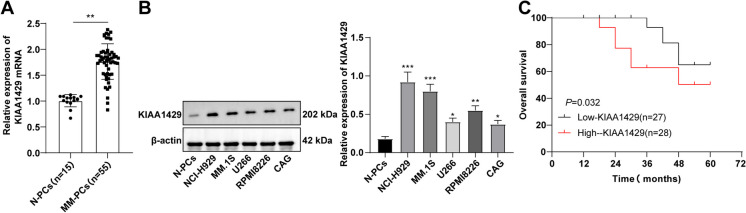
The expression abundance of KIAA1429 increases in MM patients and MM cells, and is associated with poor prognosis in MM patients. **A** RT–qPCR to detect the expression of KIAA1429 in bone marrow-derived plasma cells (MM-PCs) from MM patients and normal plasma cells (N-PCs) from bone marrow-derived healthy subjects undergoing orthopedic surgery; **B** Western blot to detect the expression of KIAA1429 in N-PCs and different MM cell lines; **C** Kaplan–Meier survival analysis to analyze the correlation between KIAA1429 mRNA levels and overall survival in MM patients. The cell experiment was repeated three times, and the data were expressed as mean ± standard deviation. The comparison between two groups was conducted using independent sample t-tests, while the comparison between multiple groups was conducted using one-way ANOVA analysis, with Tukey’s test for post-test of data. * *P* < 0.05, ** *P* < 0.01, *** *P* < 0.001

To delve into the relationship between KIAA1429 levels and MM, we compared the expression differences in MM-PCs from 55 MM patients and in N-PCs from 15 healthy subjects who underwent orthopedic surgery. RT-qPCR showed that KIAA1429 mRNA in MM-PCs was strikingly higher than that in N-PCs (*P* < 0.01, Fig. 1A). Western blot results revealed higher KIAA1429 in MM cells than in N-PCs, and the expression trend was NCI-H929 > MM.1S > RPMI8226 > U266 > CAG (all *P* < 0.05, Fig. 1B). Additionally, we classified MM patients into a KIAA1429 mRNA low expression group (< 1.80, n = 27) and a KIAA1429 mRNA high expression group (≥ 1.80, n = 28) based on the median value of KIAA1429 mRNA in MM patients. High KIAA1429 mRNA was markedly connected with reduced OS in MM patients (*P* < 0.01, Fig. 1C). In short, KIAA1429 was raised in MM and correlated with miserable prognoses, suggesting that it may be involved in MM development.

### KIAA1429 depletion curbs MM cell growth

**Fig. 2:**
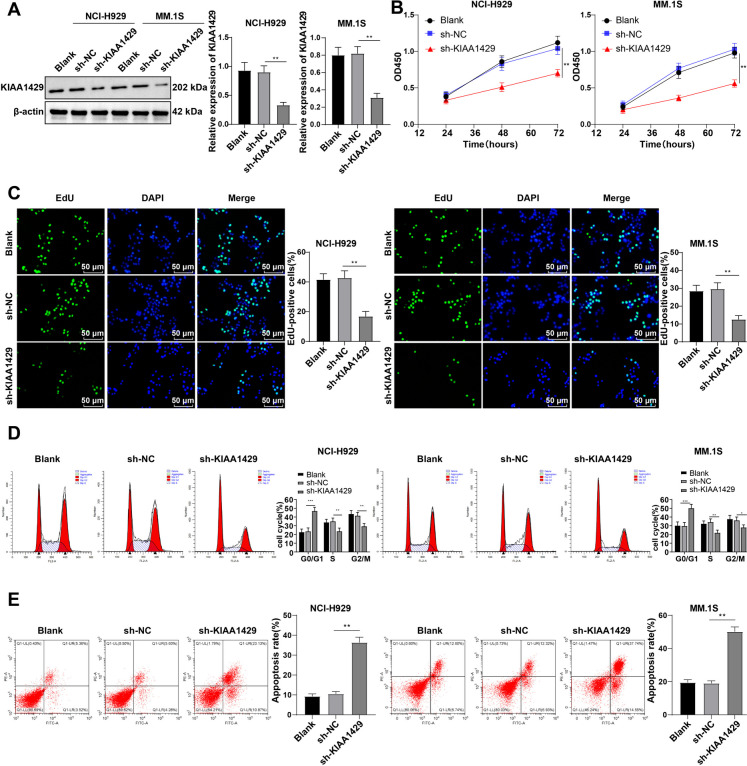
Knocking down KIAA1429 inhibits MM cell proliferation, induces G0/G1 cell cycle arrest, and promotes cell apoptosis. **A** Western blot to detect KIAA1429 expression in NCI-H929 and MM.1S cells; **B** CCK-8 method to detect cell viability; **C** EdU method to analyze cell proliferation; **D** Flow cytometry analysis of cell cycle using propidium iodide (PI) staining; **E** Annexin V-FITC/PE staining flow cytometry to analyze cell apoptosis. The cell experiment was repeated three times, and the data were expressed as mean ± standard deviation. One–way ANOVA was used for data comparison among multiple groups, and Tukey's multiple comparisons test was used for post hoc analysis; ** *P* < 0.01

NCI-H929 and MM.1S cells with relatively high KIAA1429 expression were chosen for the in vitro exploration. KIAA1429 expression was successfully knocked down by lentiviral transfection (Fig. 2A). KIAA1429 depletion notably diminished MM cell viability (Fig. 2B) and proliferation percentage (Fig. 2C). Flow cytometry demonstrated that si-KIAA1429 brought about significant aggregation of G0/G1 phase cells and significant reductions in S and G2/M phase cells (Fig. 2D) and promoted apoptosis (Fig. 2E) (all *P* < 0.01). Overall, knockdown of KIAA1429 restrained MM cell growth.

### Knockdown of KIAA1429 inhibits MM cell aerobic glycolysis

**Fig. 3:**
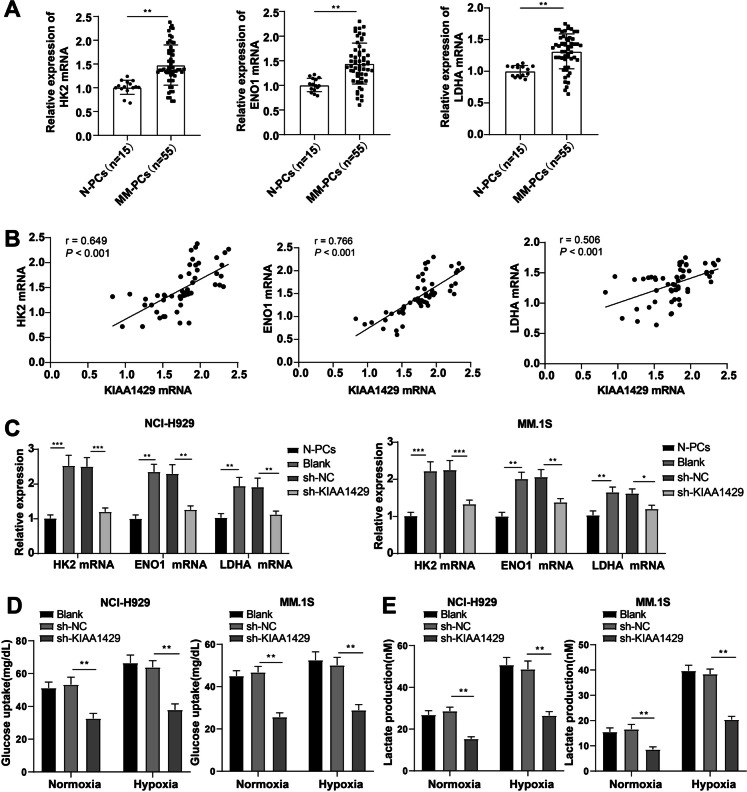
Knocking down KIAA1429 inhibits aerobic glycolysis in MM cells. **A** RT-qPCR detection of glycolytic–enhancing genes hexokinase2 (HK2), α-enolase (ENO1), and lactate dehydrogenase A (LDHA) in bone marrow-derived plasma cells (MM-PCs) from MM patients and normal plasma cells (N-PCs) from bone marrow-derived from healthy subjects undergoing orthopedic surgery; **B** Pearson method analyzed the correlation between KIAA1429 mRNA levels and HK2, ENO1, and LDHA mRNA levels in MM patients; **C** RT–qPCR to detect the mRNA levels of HK2, ENO1, and LDHA in NCI-H929 and MM.1S cells; **D** glucose uptake and **E** lactate production of NCI-H929 and MM.1S cells under normal or low oxygen (1% oxygen) conditions. The cell experiment was repeated three times, and the data were expressed as mean ± standard deviation. The comparison between two groups was conducted using independent sample t-tests, while the comparison between multiple groups was conducted using one-way ANOVA analysis, with Tukey’s test for post-test of data. * *P* < 0.05, ** *P* < 0.01, *** *P* < 0.001

Subsequently, we continued to illustrate the function of KIAA1429 on aerobic glycolysis in MM cells. RT-qPCR showed visibly higher levels of HK2, ENO1, and LDHA mRNA in MM-PCs than in N-PCs (Fig. 3A). KIAA1429 mRNA levels in MM-PCs were positively correlated with HK2, ENO1, and LDHA mRNA levels (Fig. 3B), suggesting the regulation of KIAA1429 in MM aerobic glycolysis.

RT-qPCR confirmed that these glycolysis-enhancing genes were prominently reduced in KIAA1429-silenced cells (Fig. 3C). Under normoxic or hypoxic (1% oxygen) conditions, lactate production and glucose uptake substantially diminished in si-KIAA1429 cells (Fig. 3D/E) (all *P* < 0.01). Collectively, knockdown of KIAA1429 inhibited aerobic glycolysis in MM cells.

### KIAA1429 maintains FOXM1 mRNA stability in MM cells

**Fig. 4:**
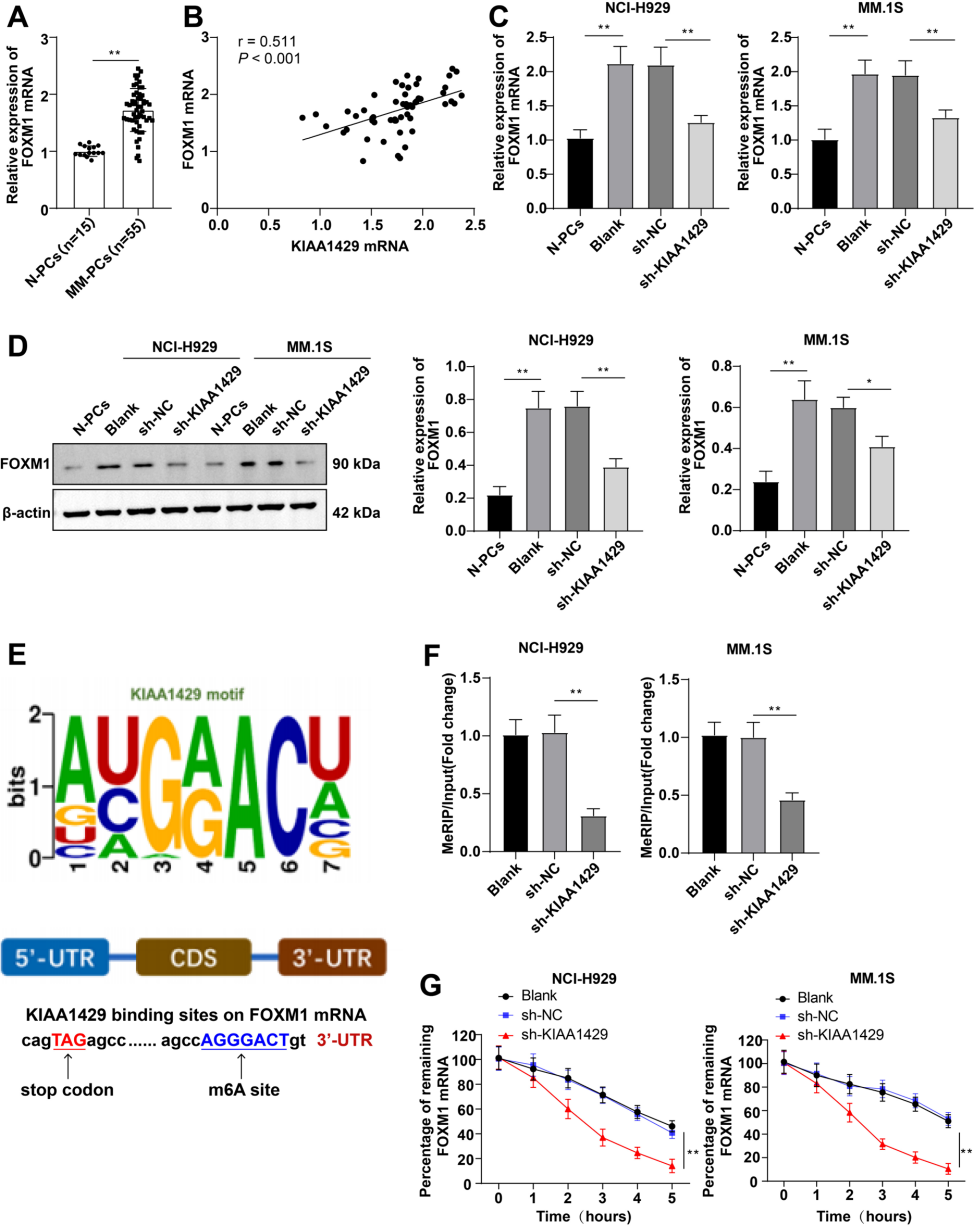
KIAA1429 maintains the stability of FOXM1 mRNA in MM cells. **A** RT–qPCR to detect the expression of FOXM1 in MM-PCs from MM patients and N-PCs from bone marrow-derived from healthy subjects undergoing orthopedic surgery; **B** Pearson method analyzed the correlation between the levels of KIAA1429 mRNA and FOXM1 mRNA in MM patients; **C** RT–qPCR and **D** Western blot to detect the expression of FOXM1 in NCI-H929 and MM.1S cells; **E** Schematic diagram demonstrated the m6A motif of KIAA1429 and the m6A site in the 3’UTR of FOXM1 mRNA (near stop codon); **F** MeRIP detection of m6A- modified FOXM1 mRNA levels; **G** RNA decay rate analysis of FOXM1 mRNA stability. The cell experiment was repeated three times, and the data were expressed as mean ± standard deviation. The comparison between two groups was conducted using independent sample t-tests, while the comparison between multiple groups was conducted using one-way ANOVA analysis, with Tukey’s test for post-test of data. * *P* < 0.05, ** *P* < 0.01

FOXM1 has been proven to be involved in regulating glycolysis in MM (Cheng et al. 2022). We further explored whether KIAA1429 could promote aerobic glycolysis in MM by stabilizing FOXM1 expression. RT-qPCR showed higher FOXM1 mRNA expression in MM-PCs than in N-PCs (Fig. 4A). KIAA1429 mRNA level in MM-PCs was visibly positively correlated with FOXM1 mRNA level (Fig. 4B). KIAA1429 knockdown greatly reduced FOXM1 levels (both *P* < 0.01, Fig. 4C/D). A m6A motif of KIAA1429 was identified in the FOXM1 coding sequence 3’UTR (Fig. 4E). MeRIP assay revealed that KIAA1429 knockdown reduced FOXM1 mRNA pulled down by m6A antibody (Fig. 4F). Additionally, the half-life of FOXM1 transcripts was markedly reduced by KIAA1429 deletion (Fig. 4G) (all *P* < 0.01). Altogether, KIAA1429 stabilizes FOXM1 mRNA through m6A modification, thereby regulating FOXM1 expression.

### KIAA1429 enhances FOXM1 mRNA stability in MM cells through YTHDF1-mediated m6A modification

**Fig. 5:**
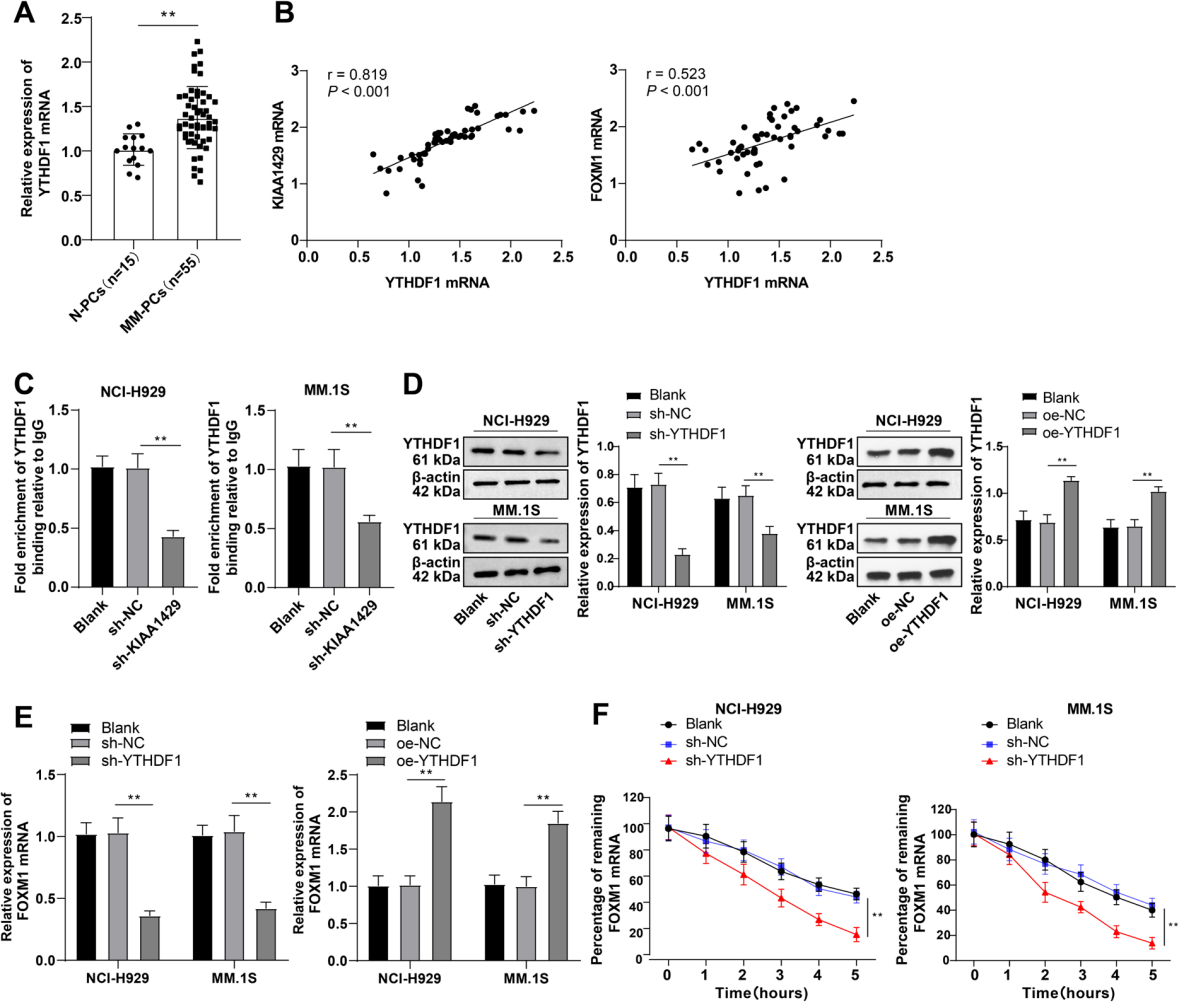
KIAA1429 enhances the stability of FOXM1 mRNA in MM cells through YTHDF1 mediated m6A modification. **A** RT–qPCR to detect the expression of YTHDF1 in MM-PCs from MM patients and N-PCs from bone marrow-derived from healthy subjects undergoing orthopedic surgery; **B** Pearson method analyzed the correlation between YTHDF1 mRNA levels and KIAA1429 and FOXM1 mRNA levels in MM patients; **C** RIP analysis of the interaction between FOXM1 mRNA and YTHDF1; **D** Western blot to detect the expression of YTHDF1 in NCI-H929 and MM.1S cells; **E** RT–qPCR to detect the level of FOXM1 mRNA; **F** RNA decay rate analysis of FOXM1 mRNA stability. The cell experiment was repeated three times, and the data were expressed as mean ± standard deviation. The comparison between two groups was conducted using independent sample t-tests, while the comparison between multiple groups was conducted using one-way ANOVA, with Tukey’s test for post-test of data. ** *P* < 0.01

YTHDF1 is one of the "readers" of m6A methyltransferases. RT-qPCR showed higher YTHDF1 mRNA in MM-PCs than in N-PCs (Fig. 5A). YTHDF1 mRNA level in MM-PCs was positively correlated with KIAA1429 and FOXM1 mRNA levels (Fig. 5B). RIP analysis found that KIAA1429 knockdown notably impaired the FOXM1 mRNA-YTHDF1 interaction (Fig. 5C), suggesting the interaction is dependent on m6A modification produced by KIAA1429. In addition, we knocked down and overexpressed YTHDF1 in MM cells (Fig. 5D). YTHDF1 knockdown substantially decreased FOXM1 mRNA levels, while YTHDF1 overexpression elevated FOXM1 mRNA (Fig. 5E). The half-life of FOXM1 was visibly reduced after YTHDF1 silencing (Fig. 5F) (all *P* < 0.01). Taken together, YTHDF1 recognizes KIAA1429-methylated FOXM1, while KIAA1429/YTHDF1 enhances FOXM1 mRNA stability in MM cells.

### Knockdown of YTHDF1 inhibits MM cell aerobic glycolysis and malignant behavior

**Fig. 6:**
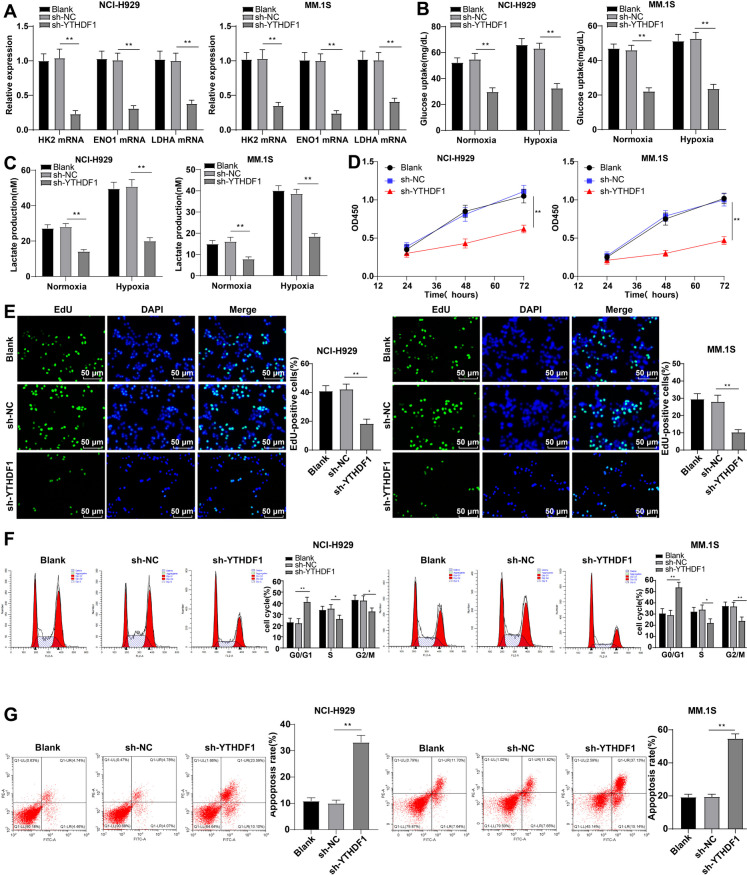
Knocking down YTHDF1 inhibits aerobic glycolysis and malignant behaviors of MM cells. **A** RT–qPCR to detect the mRNA levels of HK2, ENO1, and LDHA in NCI-H929 and MM.1S cells; **B** glucose uptake and **C** lactate production of NCI-H929 and MM.1S cells under normal or low oxygen (1% oxygen) conditions; **D** CCK-8 method to detect cell viability; **E** EdU method to analyze cell proliferation; **F** Flow cytometry analysis of cell cycle using PI staining; **G** Annexin V-FITC/PE staining flow cytometry to analyze cell apoptosis. The cell experiment was repeated three times, and the data were expressed as mean ± standard deviation. The comparison between two groups was conducted using independent sample t-tests, while the comparison between multiple groups was conducted using one-way ANOVA, with Tukey’s test for post-test of data. * *P *< 0.05, ** *P* < 0.01

We then investigate whether YTHDF1 was involved in aerobic glycolysis and malignant behavior in MM cells. YTHDF1 depletion notably diminished the levels of glycolysis-enhancing genes, glucose uptake, and lactate production (Fig. 6A-C), hindered MM cell viability and proliferation percentage (Fig. 6D/E), resulted in significant aggregation of G0/G1 phase cells, significant declines in S and G2/M phase cells, and visibly increased apoptosis (Fig. 6F/G) (all *P* < 0.01). In short, the knockdown of YTHDF1 restrained MM aerobic glycolysis and malignant cellular behaviors.

### Overexpressing FOXM1 partially restores the repressing effect of YTHDF1 depletion on MM cell aerobic glycolysis and malignant behaviors

**Fig. 7:**
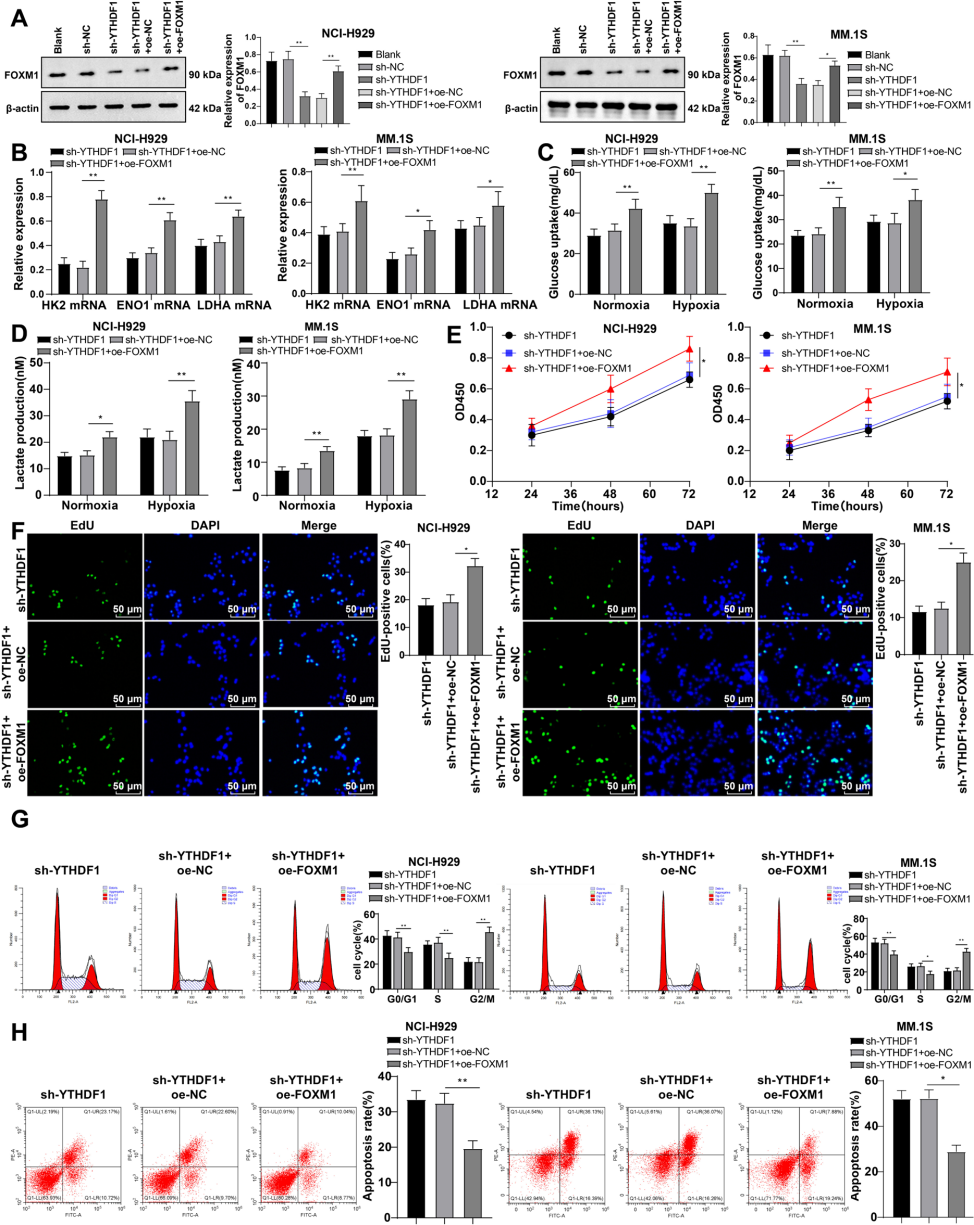
The inhibitory effect of knocking down YTHDF1 on aerobic glycolysis and malignant behaviors of MM cells can be partially restored by overexpressing FOXM1. **A** Western blot detection of FOXM1 expression; **B** RT–qPCR to detect the mRNA levels of HK2, ENO1, and LDHA in NCI-H929 and MM.1S cells; **C** glucose uptake and **D** lactate production of NCI-H929 and MM.1S cells under normal or low oxygen (1% oxygen) conditions; **E** CCK-8 method to detect cell viability; **F** EdU method to analyze cell proliferation; **G** Flow cytometry analysis of cell cycle using PI staining; (H) Annexin V-FITC/PE staining flow cytometry to analyze cell apoptosis. The cell experiment was repeated three times, and the data were expressed as mean ± standard deviation. The comparison between two groups was conducted using independent sample t-tests, while the comparison between multiple groups was conducted using one-way ANOVA, with Tukey’s test for post-test of data. * *P* < 0.05, ** *P* < 0.01

YTHDF1 was then knocked down by cell transfection while overexpressing FOXM1 for co-treatment (*P* < 0.01, Fig. 7A). The repressing effects of YTHDF1 knockdown on levels of glycolysis-enhancing genes, glucose uptake, lactate production, cell viability, and proliferation percentage in MM cells were partially nullified by overexpression of FOXM1 (*P* < 0.05, Fig. 7B-F), as well as the promotional effects on the aggregation of G0/G1 phase cells and apoptosis (*P* < 0.05, Fig. 7G/H). Collectively, knockdown of YTHDF1 inhibits aerobic glycolysis and malignant behaviors of MM cells by reducing FOXM1 reporter.

### KIAA1429 silencing hinders MM growth

**Fig. 8:**
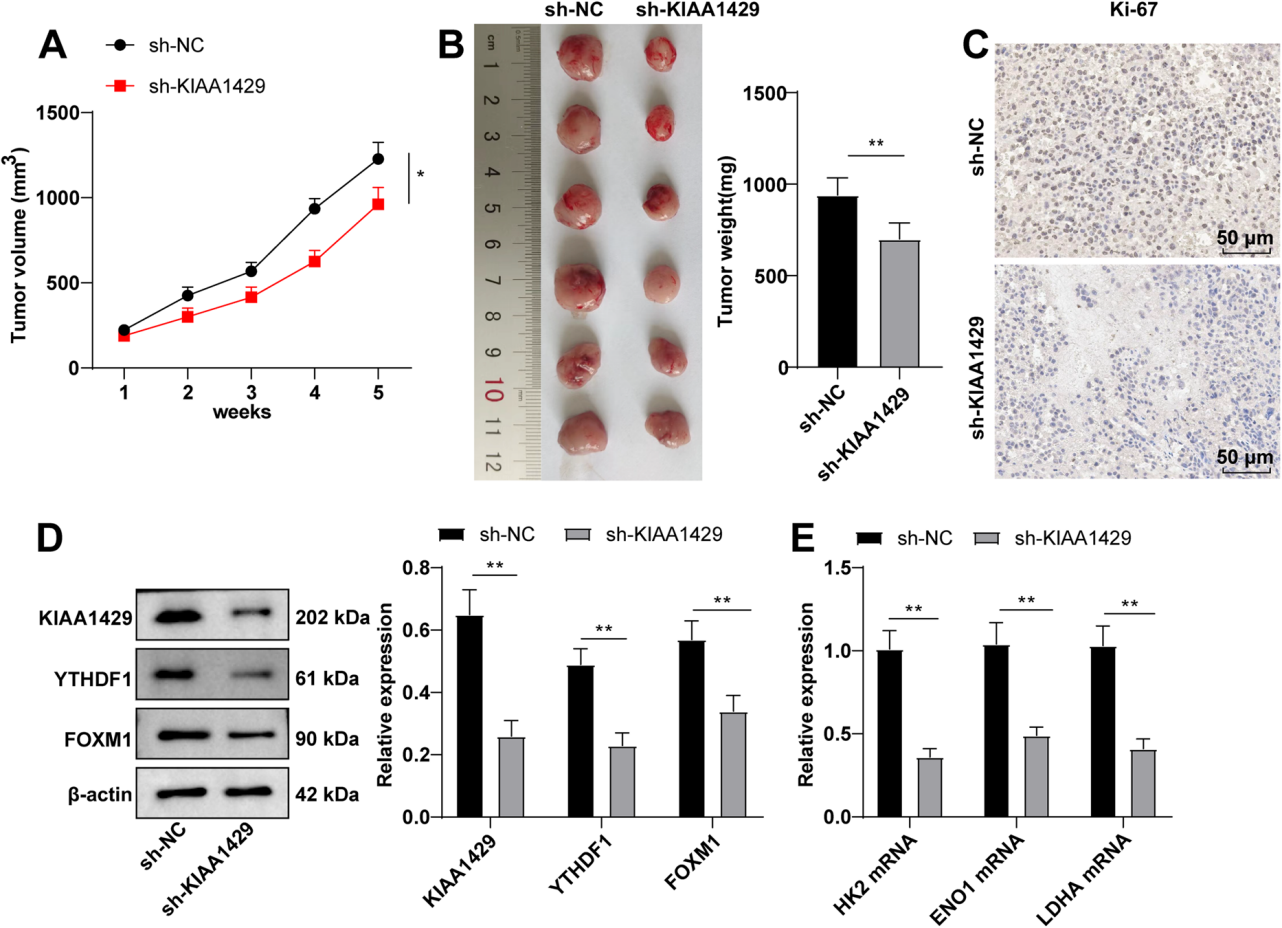
Knocking down KIAA1429 inhibits MM tumor growth in mice. **A-B** the transplantation tumor model was constructed in nude mice by subcutaneous injection of NCI-H929 cells transfected with sh-NC or sh-KIAA1429 lentivirus vector, and the volume (A) and weight (B) of mouse tumors were recorded; **C** IHC detection of the expression of tumor cell proliferation marker Ki-67; **D** Western blot to detect the expression of KIAA1429, YTHDF1, and FOXM1 in tumor tissues; **E** RT–qPCR to detect the expression of HK2, ENO1, and LDHA mRNA in tumor tissue. Animal experiment: n = 6, data were expressed as mean ± standard deviation, and the comparison between the two groups was conducted using independent sample t-test; * *P* < 0.05, ** *P* < 0.01

To validate the anti-tumor effect of KIAA1429 knockdown in vivo, we constructed transplantation tumor models by injection of NCI-H929 cells transfected with sh-NC or sh-KIAA1429 lentiviral vector. Knockdown of KIAA1429 substantially inhibited tumor growth (*P* < 0.05, Fig. 8A/B). IHC assay unveiled a significant reduction in tumor cell proliferation marker Ki-67 expression (*P* < 0.01, Fig. 8C). KIAA1429, YTHDF1, and FOXM1 levels were greatly lowered upon sh-KIAA1429 treatment (all *P* < 0.05, Fig. 8D). In addition, HK2, ENO1, and LDHA mRNA levels were strikingly reduced in tumor tissues after KIAA1429 silencing (all *P* < 0.05, Fig. 8E). Collectively, KIAA1429 silencing inhibited MM tumor growth.

## Discussion

MM bears the lion's share (approximately 10%) of all hematological malignancies; it is actually an array of distinct PC malignancies different in cytogenetics, and almost all MM patients eventually relapse (Rajkumar 2019). Over the past few decades, research has confirmed aerobic glycolysis as an attribute of tumor cells and regulator of tumor microenvironment (Gu et al. 2017). Inhibition of aerobic glycolysis has been validated to overcome melphalan resistance in MM cells (Zub et al. 2015). Thereby, we also focused on aerobic glycolysis to find new targets for MM therapy. KIAA1429 is a pivotal m6A methyltransferase with crucial roles in cancers, showing tumorigenic functions in various cancers (Zub et al. 2015). Several articles have unveiled the promoting function of KIAA1429 on aerobic glycolysis in different tumors (Xu et al. 2023a, b; Yang et al. 2021). However, the expression pattern and regulatory property of KIAA1429 in MM is largely unidentified. This paper illustrated the mechanism of KIAA1429 in MM and uncovered that KIAA1429 knockdown could reduce FOXM1 expression through YTHDF1-mediated m6A modification, thus repressing MM aerobic glycolysis and tumorigenesis.

Many studies have unveiled the high expression of KIAA1429 in tumor tissues and its correlation with prognosis and tumorigenesis (Lan et al. 2019). For instance, KIAA1429 was at higher levels in hepatocellular carcinoma, and patients with higher KIAA1429 levels had poor OS (Lan et al. 2019). Higher levels of KIAA1429 were also found in lung adenocarcinoma and its oncogenic role was figured out in an m6A‐YTHDF2 way (Zhang et al. 2022). Nonetheless, only the research by Wang et al. initially revealed the role of KIAA1429 in the prognosis of MM by analyzing the gene expression profiles of 21 m6A regulators based on data from the public database and through clinical analysis (J. Wang et al. 2023), and KIAA1429 levels in MM samples have not been measured. Based on these studies on KIAA1429 levels in other cancers we speculated that KIAA1429 might also be upregulated in MM. In the present research, KIAA1429 expression was therefore tested in MM-PCs and MM cells and its correlation with OS of MM patients was analyzed, which revealed higher levels of KIAA1429 in MM-PCs and MM cells and the correlation of KIAA1429 upregulation with reduced OS of MM patients. Innovatively, our study further ascertained the oncogenic action and mechanism of KIAA1429 in MM through cell experiments. The results unraveled that KIAA1429 knockdown inhibited MM cell proliferation, promoted apoptosis, and repressed aerobic glycolysis. Despite no studies on the specific role and mechanism of KIAA1429 in MM, multiple studies have disclosed that KIAA1429 accelerates aerobic glycolysis and carcinogenesis in other cancers, like gastric cancer (Yang et al. 2021), oral squamous cell carcinoma (Xu et al. 2023a, b), and colorectal cancer (Li et al. 2022) in a m6A-dependent manner. Accordingly, we then focused on the m6A modification of KIAA1429 in MM.

m6A levels decrease strikingly in cells upon KIAA1429 knockdown, indicating the significant role of KIAA1429 in m6A modification (Yue et al. 2018). Of particular note, the role of KIAA1429 upregulating FOXM1 through m6A modification has been observed only in gastric cancer (Tang et al. 2023; Zhu et al. 2022). Additionally, YTHDF1 was revealed to enhance FOXM1 levels via m6A in BC (Chen et al. 2022). Intriguingly, our study first demonstrated that KIAA1429 elevated FOXM1 mRNA expression and stability in MM cells via YTHDF1-mediated m6A modification. Notably, although YTHDF2 was exhibited to foster MM cell proliferation (Liu et al. 2023), no research has probed the expression and role of YTHDF1 in MM. Prior research revealed upregulation of YTHDF1 and its association with the prognosis in other cancers, like melanoma (T. Li et al. 2020; Wang et al. 2022). Our results unveiled higher levels of FOXM1 and YTHDF1 mRNA in MM-PCs and positive correlations of KIAA1429 levels in MM-PCs with FOXM1 and FOXM1 levels. YTHDF1 is a cancer driving factor that can drive tumorigenicity and metastasis by promoting glycolysis (Yao et al. 2022). METTL3 maintained HK2 stability via YTHDF1-mediated m6A, driving Warburg effect in cervical cancer (Wang et al. 2020). YTHDF1 silencing, consistent with the effect of KIAA1429 silencing, downregulated glucose uptake and lactate production in oral squamous cell carcinoma (Xu et al. 2023a, b). Furthermore, concordant with the properties of KIAA1429 depletion, our further experimental data validated that YTHDF1 knockdown inhibited aerobic glycolysis and malignant episodes of MM cells. FOXM1 transcriptionally regulates genes associated with m6A modification and glycolysis (Xu et al. 2023a, b). FOXM1 upregulation raised LDHA levels, glucose utilization, and lactate production and facilitated pancreatic cancer cell growth (Cui et al. 2014). FOXM1 enhances glucose uptake and oxygen consumption and predicts poor survival in relapsed MM (Cheng et al. 2022). Innovatively, our results elucidated that the repressing effect of knocking down YTHDF1 on MM aerobic glycolysis and malignant behaviors were partially restored by overexpressing FOXM1. Moreover, the animal experiments further confirmed that KIAA1429 silencing diminished levels of YTHDF1, FOXM1, HK2, ENO1, and LDHA and suppressed tumor growth.

The limitation of this article lies in the insufficiency of animal experiments and clinical validation. Due to the limitations of the experimental period and samples, we were not able to include any more MM patients for the survival analysis. We will conduct multi-center studies to validate the link between high KIAA1429 expression and the poor prognosis of MM by including more clinical cases. The other regulatory pathways of KIAA1429 involved in aerobic glycolysis and development during MM still need to be studied. We will conduct more research to address the above shortcomings in the future.

Of course, this article had several novelties because the existing studies on KIAA1429, FOXM1, and YTHDF1 either focused on other tumors or did not establish a complete validation of the mechanism. Initially, the expression patterns of KIAA1429 and YTHDF1 were firstly tested in MM-PCs and MM cells. Next, the action of KIAA1429 and YTHDF1 in aerobic glycolysis and carcinogenesis during MM was analyzed for the first time. Finally, the mechanism of KIAA1429/FOXM1/YTHDF1 was firstly discovered in MM.

In summary, our paper elucidated that knocking down KIAA1429 can reduce FOXM1 expression through YTHDF1-mediated m6A modification, thereby inhibiting MM aerobic glycolysis and inhibiting tumor development. These findings offer a potential therapeutic option for MM via KIAA1429.

## Supplementary Information

Below is the link to the electronic supplementary material.

Supplementary file1 (DOCX 16 KB)

## Data Availability

The datasets generated during and analyzed during the current study are not publicly available, but are available from the corresponding author on reasonable request.
