# Supplementary material for: KIAA1429 increases FOXM1 expression through YTHDF1–mediated m6A modification to promote aerobic glycolysis and tumorigenesis in multiple myeloma
Source: Cell Biol Toxicol. 2024 Jul 26;40(1):58. doi: 10.1007/s10565-024-09904-2 (PMC11282141; doi:10.1007/s10565-024-09904-2)
Supplement: Supplementary file 1 — Supplementary file1 (DOCX 16 KB) [file 10565_2024_9904_MOESM1_ESM.docx]

**Graphical Headlights**

1. KIAA1429 upregulation is observed in MM patients and cells and is associated with poor prognosis in MM patients.

2. KIAA1429 knockdown inhibits aerobic glycolysis and cell proliferation and promotes apoptosis in MM.

3. KIAA1429 enhances FOXM1 mRNA stability through YTHDF1-mediated m6A modification in MM.

4. YTHDF1 knockdown represses aerobic glycolysis and malignant behaviors of MM cells by downregulating FOXM1.
